# Age and QMP exposure affect the nutritional preferences of caged *Apis mellifera* worker honeybees

**DOI:** 10.1007/s13592-026-01246-8

**Published:** 2026-01-26

**Authors:** Anthony Bracuti, Zoe Lois Hudson, Emily Hazel Pidcock, Kane Yoon, Elizabeth Jenness Duncan

**Affiliations:** School of Biology, Faculty of Biological Sciences, University of Leeds, Leeds, LS2 9JT UK

**Keywords:** Honeybee, Nutrition, Pheromone, QMP, Feeding

## Abstract

Eusocial insect queens often use pheromones to prevent reproduction in the worker caste, enforcing the reproductive constraint that is central to eusociality. In *A. mellifera* honeybees, the queen emits several pheromones that affect worker reproduction, the most important being QMP. Although the effects of QMP have been studied in some detail, the mechanisms by which it brings about reproductive constraint in workers are still unclear. Remarkably, QMP is also able to repress reproduction in other insects, including the fruit fly *Drosophila melanogaster*, in which QMP has been shown to induce a starvation-like response. Here we use caged newly eclosed workers with an ad libitum choice of protein and sugar food sources to investigate whether QMP alters dietary intake in the honeybee. We show that initially, irrespective of QMP exposure, workers only consume protein, before shifting to carbohydrate after 4 days. We also show that QMP exposure results in an increased preference and intake of carbohydrates in worker bees, raising the possibility that QMP also induces a starvation-like response in honeybees.

**Supplementary Information:**

The online version contains supplementary material available at 10.1007/s13592-026-01246-8.

## Introduction

 The defining feature of eusociality is the reproductive division of labour (Wilson 1971). In *A. mellifera* honeybees, this is maintained in part by the presence of pheromones produced by the queen (Princen et al. 2019), particularly queen mandibular pheromone (QMP), which suppresses the reproduction of workers by preventing the activation of their ovaries (Hoover et al. 2003). QMP is not the only pheromone to mediate reproductive constraint in this species; however, several other compounds (Mohammedi et al. 1998; Maisonnasse et al. 2010) and queen pheromones (Wossler and Crewe 1999; Princen et al. 2019) are also able to bring about reproductive constraint, indicating a high degree of redundancy in this eusocial regulatory mechanism (Princen et al. 2019).

QMP, produced in the mandibular glands of queens (Slessor et al. 1990), is comprised of five main compounds (Slessor et al. 1990; Plettner et al. 1996, 1997). In addition to inhibiting reproduction, QMP also produces other effects in the honeybee worker, including inducing care behaviours (Fischer and Grozinger 2008), regulation of swarming (Winston et al. 1989), inhibiting rearing of queens (Pettis et al. 1997), and inducing retinue behaviour (Slessor et al. 1988). Despite the large body of research investigating the different functions of this pheromone, the mechanism of action for QMP’s repressive effect on worker reproduction is not fully understood at a physiological, or molecular, level.

QMP is also able to bring about the repression of reproduction in other, phylogenetically diverse, species including the bumblebee *B. terrestris* (Princen et al. 2020), and the fruit fly *D. melanogaster* (Camiletti et al. 2013). With the latter species being almost 370 million years diverged from *A. mellifera* (Misof et al. 2014). Work in *D. melanogaster* has shown that QMP induces a starvation-like response, possibly producing reproductive repression as a by-product of starvation-induced diapause (Lovegrove et al. 2023). This would possibly indicate that QMP may have evolved to inhibit reproduction in honeybee workers via sensory exploitation of highly conserved pathways, as previously suggested (Oi et al. 2015). An example of a target of this sensory exploitation might be Notch signalling in QMP-mediated reproductive repression in honeybee workers, which has been shown to be activated by the presence of QMP (Duncan et al. 2016). In this scheme, the highly conserved Notch signalling pathway may have been coopted to induce reproductive constraint in worker honeybees in a way which also results in reproductive constraint in those phylogenetically diverse species.

Historically, investigations of QMP activity on the various aspects of honeybee behaviour and physiology have been carried out both within a native hive environment (*in alvo*, e.g. (Pankiw et al. 1994)), and in more sterile environments in cages in laboratory settings (*in cavea*, e.g. (Pirk et al. 2010)). These *in cavea* experiments allow for the strict control of extraneous variables which could impact the phenotype being investigated (for example, the presence of other pheromones produced by the queen, or developing brood), but they may also produce workers that are not entirely biologically equivalent to those reared under normal in-hive (*in alvo*) conditions. These *in cavea* studies also require the artificial supplementation of food. Different studies have used diverse feeding regimens (Williams et al. 2013), ranging from a relatively natural sugar fondant/pollen setup (Mohammedi et al. 1998) to a protein-heavy complete bee food (CBF, used to maximally induce ovary activation) (Duncan et al. 2016, 2020).

In colonies, young workers perform nursing and brood-care tasks which require pollen (Crailsheim 1990; Robinson 1992), whereas older foragers consume nectar to fuel flight (Crailsheim 1990). There has been some investigation into the preference of honeybee workers for different food types, such as the preferences of honeybees towards more metabolisable forms of protein (Pernal and Currie 2000; Pirk et al. 2010). Food preference and nutrient intake therefore vary with worker behavioural role and physiological state. Several pheromones have been shown to affect these feeding dynamics; for example, (E)-β-ocimene produced by brood simulates foraging and brood care (Maisonnasse et al. 2010; He et al. 2016), while QMP alters lipid metabolism and fat body composition (Fischer and Grozinger 2008; Corby-Harris et al. 2022) as well as protecting against starvation (Fischer and Grozinger 2008). However, the relationship between QMP exposure, feeding preferences, and diet consumption has not been directly examined *in cavea* conditions. This study aimed to investigate the effect of QMP exposure on feeding preferences *in cavea* for queenless *A. mellifera* workers, as well as testing the hypothesis that, similarly to *D. melanogaster* fruit flies, QMP induces starvation-like behaviour in worker honeybees by, for example, increasing the amount of food being eaten.

## Methods

### Honeybee husbandry

Polystyrene national-type hives of honeybees were kept at the University of Leeds, with standard beekeeping practice. Colonies were fed sugar fondant (BeeCandee, Beekeeping Supplies UK) during winter and spring and pollen cake (ApiCandy, Beekeeping Supplies UK) during the early spring.

For experiments, frames of eclosing brood were taken from multiple queen-right hives over the summers (May–September) of 2023 and 2024.

### *In cavea* experiments

Brood frames from the hives were emptied of adult bees and placed into a 35 °C incubator for up to 24 h. All the workers which eclosed in this time were mixed, and 100 of these bees were randomly assigned to metal cages (10 cm × 10 cm × 5.5 cm steel with removable glass front and air holes, www.small-life.co.uk). The caged bees were kept in the dark at 35 °C, fed ad libitum sugar fondant (3:1 ground table sugar to honey by weight), pollen cake (7:3 ground pollen supplied from LiveMoor to honey by weight), and water, refreshed every 24 h, recording consumption of each food type.

Each cage was provided treatment in the form of queen pheromone or solvent control (ethanol) every 24 h. QMP was provided as a 20 µl aliquot of 0.1 Queen equivalent per day (Qe; 1 Qe is the amount of pheromone produced in a day by a single queen: 261.8 µg ODA, 104.7 μg HDA of both enantiomers combined, 26.2 µg HOB, and 2.62 µg HVA (Pankiw et al. 1996), supplied by Intko Supply Ltd., Canada) in ethanol on a microscope slide on the bottom of the cage, with the slide replaced every 24 h. Dead bees were also removed, and deaths recorded, every 24 h.

After 10 days, all remaining bees were dissected to remove their ovaries, which were analysed to confirm QMP-mediated repression of workers. Some cages were taken through to day 20; however, high mortality rates made this data unreliable, and so it was censored.

### Statistics

Graphs were produced in R using the ggplot2 (Wickham 2016) package and finished in Microsoft PowerPoint. For the consumption graph, means of each average consumption for each day were calculated and standard deviation was used for error bars. For the Cohen’s D graph, Cohen’s D values were calculated measuring the effect size between fondant consumption by treatment for each day, with the error bars representing the upper and lower limits.

All analysis was performed in R (R Core Team 2021): The difference of food given to the bees and food removed from the bees 24 h later for each cage was calculated into a feeding difference value for each of fondant and pollen. This value was then used to do individual pairwise comparisons between each of the treatments for each day via GLM using a distribution determined via the descdist package from the fitdistrplus package (Delignette-Muller and Dutang 2015) in R. For Gaussian fitted models, an ANOVA was performed using an *F*-test, while for the gamma fitted models, a Log-Rank test was used to generate significance values. When these were significant, post hoc comparisons were undertaken using a Sidak adjustment for multiple comparisons at a given time point.

Overall significance of treatment effect on food consumption was also calculated using the data aggregated across all days, using a GLM with Gaussian distribution. Cage was initially introduced as a covariate, but was found not to significantly predict consumption difference, and so was excluded. The data distribution was determined using the descdist function from the fitdistrplus package (Delignette-Muller and Dutang 2015) in R. Significance was determined using ANOVA with *F*-test, followed by Sidak post hoc adjustments as described above.

## Results

### Food preferences switch from protein-rich food to carbohydrate rich food

Over the course of two summers, a total of 70 ethanol and 62 QMP cages were investigated, and their food intake (either fondant or pollen) was recorded daily.

**Figure. 1:**
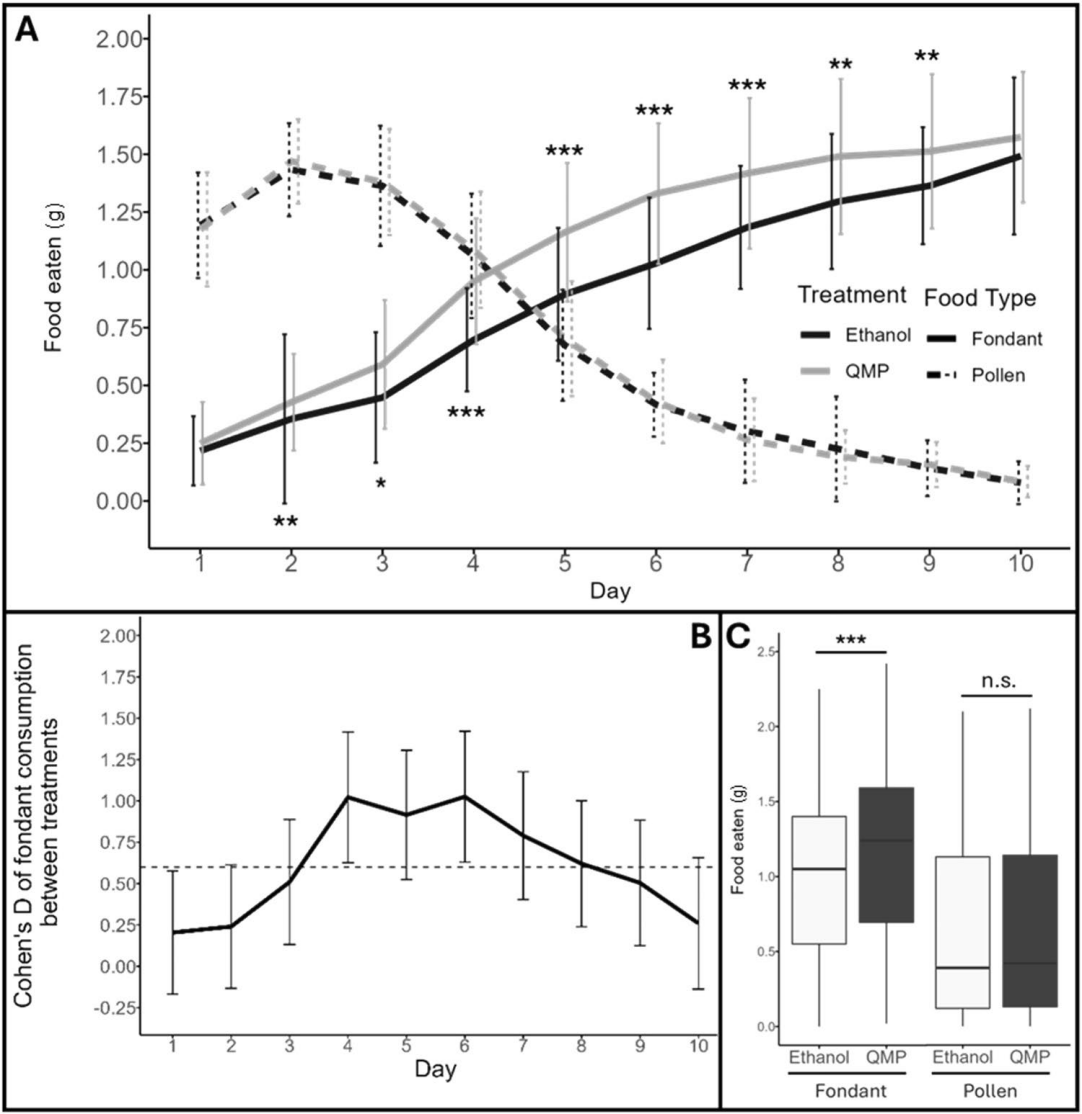
Different food types consumed by queenless worker *A. mellifera* honeybees reared *in cavea* in the presence and absence of queen mandibular pheromone. The consumption of two food sources, sugar fondant (solid lines) and pollen cake (dashed lines), was measured each day for 10 days for each of two treatments: 0.1 Qe of QMP per day (grey lines) or ethanol solvent control (black lines). In **A**, the mean value is plotted for both treatments and food types with error bars representing one standard deviation; significance is given as *P < 0.05, **P < 0.01, ***P < 0.001, calculated via glm with post hoc Sidak adjustment. In **B**, the Cohen’s D of effect size between treatments of fondant consumption from panel **A** is shown, with the dashed line showing a value of 0.6, the threshold between a medium and large effect size. In **C**, the cumulative food consumption is shown for each treatment and food type; significance is given as n.s. = *P* > 0.05; *** = *P* < 0.001; calculated via glm with post hoc Sidak adjustment.

As seen in Figure 1A, irrespective of treatment, newly eclosed workers initially prefer protein-rich pollen cake, before a switch of preference to the carbohydrate-rich sugar fondant occurring during the fifth day after eclosure. By day 10, the consumption of pollen cake falls to almost zero. This is consistent with previously published research showing the initial importance of protein-rich food in the days immediately after eclosure (Pernal and Currie 2000; Pirk et al. 2010).

### QMP-exposed worker bees consume more fondant than those exposed to solvent control

QMP has no effect on the consumption of protein (in the form of pollen cake) (*F* = 0.0908, df = 1316, *P* = 0.7673).

However, when exposed to QMP at a concentration of 0.1 Qe per day, honeybee worker consumption of carbohydrates (in the form of sugar fondant) exceeds that of bees exposed to solvent controls (*F* = 28.745, df = 1315, *P* < 0.001). This difference is statistically significant from days two to nine after eclosion, with the greatest effect size occurring from days four to six (Table I and Figure 1A, B).

**Table I:** Results of the statistical tests investigating the differences in fondant consumption by worker honeybees exposed to QMP and solvent control

Day	Residual degrees of freedom	Residual deviance	Adjusted *P*-value	Cohen’s D effect size
1	112	2.989	0.2788	0.20
2	112	46.115	0.0028	0.24
3	112	35.011	0.0102	0.51
4	112	11.681	< 0.001	1.02
5	112	9.651	< 0.001	0.92
6	112	9.663	< 0.001	1.03
7	112	9.804	0.001	0.79
8	112	10.997	0.0013	0.62
9	112	9.585	0.0086	0.50
10	112	9.778	0.1959	0.26

When observing total food consumed per cage, the statistically significant difference in overall fondant consumption between treatments, but not pollen consumption, can be clearly seen (Figure 1C; fondant: *F* = 28.745, df = 1315, *P* < 0.001; pollen: *F* = 0.0908, df = 1315, *P* = 0.7633).

For each biological replicate, bees from QMP-exposed cages and solvent-only control cages were dissected on day 10 to assess ovarian activity. In all cases, QMP exposure resulted in statistically significant repression of ovary activity compared with the ethanol-only solvent (Supplementary Fig. 1).

Interestingly, workers provided with fondant alone did not activate their ovaries, regardless of QMP exposure (Supplementary Fig. 2). Bees fed only pollen exhibited significantly lower survival (Supplementary Fig. 3), whereas those fed either pollen plus fondant or fondant alone showed significantly higher survival (Supplementary Fig. 3).

## Discussion

This study aimed to investigate whether QMP alters the nutritional preference of newly eclosed worker honeybees. Building on previous work in *D. melanogaster*, where QMP induces a starvation-like response (Lovegrove et al. 2023), we hypothesised that QMP might similarly influence feeding behaviour in honeybees. Our findings support this hypothesis, but only for carbohydrate consumption. QMP exposed workers showed a significant and sustained increase in carbohydrate-rich (fondant) consumption while protein (pollen-cake) intake remained unaffected (Figure 1).

That QMP exposure results in an increase in sugar consumption is perhaps counterintuitive. Given that QMP-exposed bees are less reproductively active (and therefore devoting fewer metabolic resources to egg production), the energy requirements within these bees should theoretically be lower, all else being equal (Wigglesworth 1960). Similarly, we would expect to see those bees which are more reproductively active to have higher protein needs, due to the role of metabolic protein in vitellogenin synthesis (Izumi et al. 1994; Wu et al. 2021). The lack of difference in pollen consumption (the only protein source for honeybees in general, and particularly in the cages, though there are trace amounts of amino acids in the honey used in the sugar fondant) is therefore surprising and indicates that the effect of QMP on food consumption is likely unrelated to reproduction directly.

The increase in consumption of fondant under QMP exposure suggests that QMP may be triggering a shift in perceived nutritional state or metabolic demand, consistent with a starvation-like response, despite the bees being in a controlled nutrient abundant environment, as was seen for *D. melanogaster*. Interestingly, nutritional state modulates workers’ responsiveness to QMP (Walton et al. 2018), further suggesting that diet and pheromonal signalling interact closely in the honeybee, potentially acting through shared or overlapping physiological pathways.

The increase in consumption of carbohydrates might also reflect a QMP-induced increase in metabolic activity, possibly indicating a change in physical activity which necessitates the increase in metabolism and therefore sugar consumption. However, the presence of a queen has been shown to have a calming effect on workers (Grodzicki et al. 2020), and it has been shown that QMP reduces activity in workers (Beggs et al. 2007), although this latter study used much higher QMP exposures than in this study, and better techniques for quantifying physical activity have since been developed (Chiara and Kim 2023). It is worth applying these techniques to bees reared under the conditions presented here, in order to confirm the effect that QMP has on activity.

Notably, QMP is known to inhibit “social-aging”, whereby the innate age-based polyethism of honeybees is delayed, resulting in less foraging activity (Pankiw et al. 1998). This would imply that QMP should decrease sugar consumption, due to the lower anticipated metabolic requirements associated with non-foraging activities as foraging requires higher energy expenditure to sustain flight (Casey 1981). However, confirming this would require additional data measuring physiological proxies for social aging (e.g. changes in haemolymph vitellogenin titres (Nakaoka et al. 2008) or fat body lipid and protein levels (Bertholf 1925)).

It is also important to note that food intake patterns in caged workers are likely to differ from those in colony conditions, where foragers, for example, require more nutrients to sustain flight. In our caged setup, where brood and flight activity are absent, the increased carbohydrate consumption observed in QMP-exposed workers may reflect a shift toward a more nurse-like metabolic state. The increase in fondant consumption we observed is consistent with the increased lipid storage in the fat body that comes about as a result of nursing behaviours in honeybees (for royal jelly production in the hypopharyngeal glands) (Crailsheim et al. 1992; Toth and Robinson 2005). This pattern is similar to that reported by Corby-Harris et al. (2022), where exposure of young bees to QMP resulted in altered fat body composition (increased lipid and decreased protein) (Corby-Harris et al. 2022), supporting the idea that QMP influences nutritional metabolism as well as reproductive state.

It is possible that QMP is able to bring about repression of worker reproduction and increased sugar consumption via the role of adult diapause mechanisms in honeybees. The role of diapause in QMP-mediated repression of reproduction in *D. melanogaster* has been postulated (Knapp et al. 2022), whereby QMP has evolved to coopt ancestral diapause mechanisms to bring about reproductive repression in that species. A similar diapause-like dormancy mechanism exists in the honeybee as the winter phenotype, whereby during winter, reproduction is switched off in queens, but also in workers (Seeley and Visscher 1985; Knoll et al. 2020), combined with a host of other metabolic, genetic, and behavioural changes (Phillips and Demuth 1914; Rockstein 1950; Owens 1971; Bresnahan et al. 2022). Interestingly, recent work has shown that worker exposure to QMP components varies seasonally but does not affect retinue size (Carroll et al. 2023). This suggests that although QMP levels vary across the year, its behavioural effects may remain stable. The influence of QMP on winter workers is an interesting area for future studies. Notably, this adult reproductive diapause is distinct from the larval diapause brought about by nutrient stress that occurs in many insects (Hahn and Denlinger 2011). It is possible that, as is suggested in the fruit fly, in *A. mellifera*, QMP acts to induce elements of this adult diapause to prevent worker ovary activation.

It is possible that QMP’s ability to repress reproduction in adult worker honeybees under summer conditions is a co-option of the seasonal mechanisms which bring about the repression of worker reproduction under winter conditions and that a secondary effect of this coopted mechanism is the winter phenotype’s propensity to consume food as necessary for maintaining temperature homeostasis in the winter cluster (Owens 1971). The increased consumption in carbohydrates (but not pollen) would therefore be a side effect of QMP-mediated reproductive constraint.

Regardless of the reason for increased consumption of carbohydrates, the fact that the overconsumption of food under QMP-exposed conditions is similar between *A. mellifera* and *D. melanogaster* potentially demonstrates that they are bringing about reproductive constraint via the same mechanism.

## Supplementary Information

Below is the link to the electronic supplementary material.

ESM 1 (PDF 257 KB)

## Data Availability

The data that support the findings of this study are available from the author upon reasonable request.
