# Supplementary material for: Age and QMP exposure affect the nutritional preferences of caged Apis mellifera worker honeybees
Source: Apidologie. 2026 Jan 26;57(1):8. doi: 10.1007/s13592-026-01246-8 (PMC12835030; doi:10.1007/s13592-026-01246-8)
Supplement: Supplementary file 1 — (PDF 257 KB) [file 13592_2026_1246_MOESM1_ESM.pdf]

Title:

Age and QMP exposure affect the nutritional preferences of *Apis mellifera* worker honeybees

Supplementary Data

Journal:

Apidologie

Authors:

Anthony Bracuti[1]: Anthony.bracuti@gmail.com

Zoe Lois Hudson[1]: bs19zlh@leeds.ac.uk

Emily Hazel Pidcock[1]: emilyhazelpidcock@gmail.com

Kane Yoon[1]: kanejyoon@gmail.com

Elizabeth Jenness Duncan[1*]: e.j.duncan@leeds.ac.uk

1. School of Biology, Faculty of Biological Sciences, University of Leeds, LS2 9JT Leeds, United Kingdom.

* Corresponding author e.j.duncan@leeds.ac.uk

QMP-exposure produces ovary repression in all cages

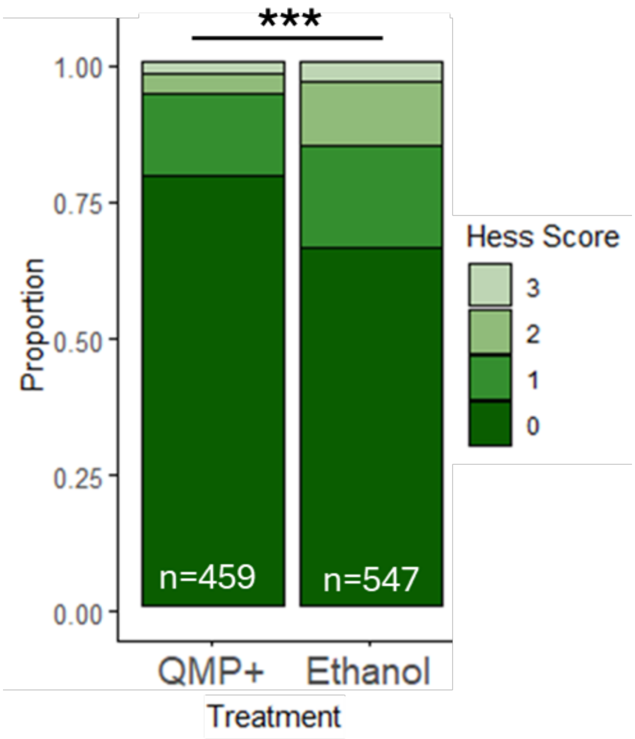

*Figure 1 – Honeybee workers exposed to QMP from eclosure, show repressed ovaries when compared with bees not exposed to QMP, as measured by a modified Hess score (3 = fully developed ovum present; 2 = egg yolk visible; 1 = cell differentiation between ovum and nurse cells visible; 0 = no cell differentiation visible). The x-axis shows treatment (QMP at 0.1 Qe per day provided as liquid on microscope slide, or ethanol solvent control), while the y-axis shows proportion of workers at each Hess score, shown in the legend. Significance was calculated using CLMM with post-hoc tukey-adjusted test; *** = P<0.001.*

In Figure 1, an example of a given experiment, in which six cages of each treatment are analysed for ovary activity via a modified Hess score. Similar analyses were undertaken for all experiments from which the 149 cages were taken to investigate food choice and consumption, in which all experiments indicated a significant difference in ovary activity between QMP and ethanol-only exposed worker honeybees. The overall activation was different between experiments, due to differences in origin of worker honeybees and different times of year, and so each assay was always carried out with both QMP and ethanol-only controls in order to demonstrate the efficacy of the QMP.

Honeybee workers fed only fondant do not activate their ovaries

As can be seen in Figure 2, if fed an *ad libitum* pollen and sugar fondant diet (FandP), workers activate their ovaries (though much less so if also exposed to QMP). If only fed fondant (i.e. without pollen, or any other protein source), workers do not activate their ovaries regardless of pheromone

exposure. Fondant or FandP diet has no effect on mortality in the 10-day period of this assay, however bees only fed pollen, with no fondant do not survive past day 9 (Figure 3)

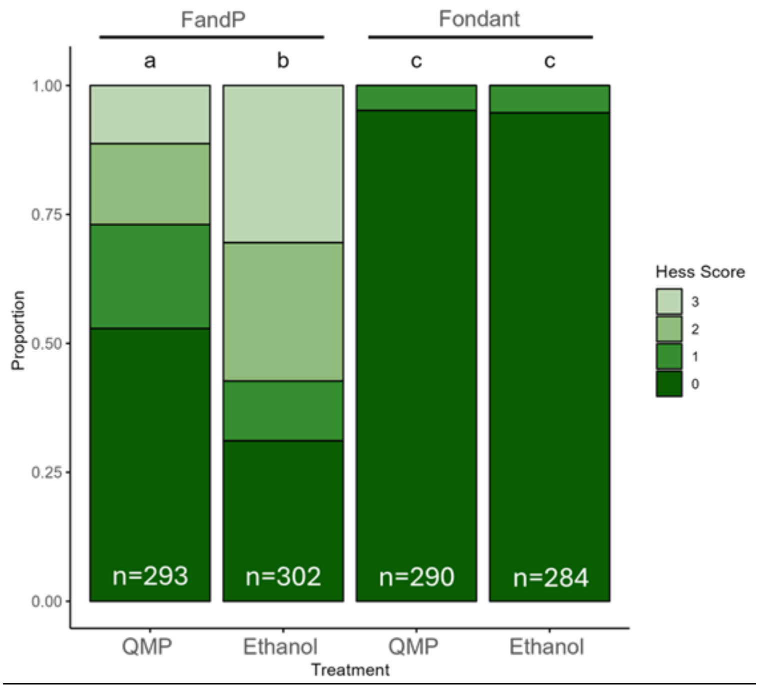

Figure 2 - Showing a proportional stacked bar chart of ovary activity of A. mellifera workers when fed different quality foods. *Ovary activity was measured via modified Hess score (0 = inactive ovaries, 1 = cell differentiation present, 2 = yolk deposition present, 3 = fully developed ovum present). The y-axis shows proportion of ovaries of a given Hess score, while the x axis shows treatment of either 0.1Qe QMP per day or ethanol solvent control, or food types of either FandP diet, or fondant only. Significance given as letters (P<0.05) calculated via CLMM and post-hoc tukey pairwise test. Under the FandP diet, QMP is able to repress the activity seen in the ethanol control, but under fondant diet, no activation of ovaries occurs.*

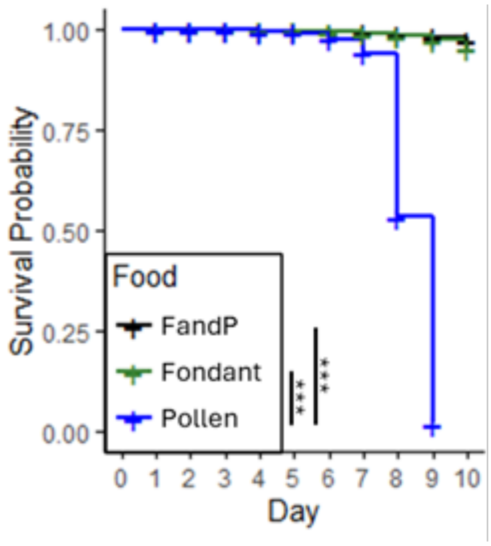

Figure 3 – Showing a Kaplan-Meier survival curve of honeybee workers which have been fed different food sources. *The y-axis shows survival probability and the x-axis day of experiment, food is given as colour, where "fondant" is fondant only, "pollen" is pollen only and "FandP" is a choice of either. Bees fed only pollen could not survive to the end of the experiment, but there is no difference in survival between the other two food types.*
